# Are We Ready for Newborn Genetic Screening? A Cross-Sectional Survey of Healthcare Professionals in Southeast China

**DOI:** 10.3389/fped.2022.875229

**Published:** 2022-05-06

**Authors:** Xian Wu, Yuqi Yang, Lingna Zhou, Wei Long, Bin Yu

**Affiliations:** Changzhou Maternal and Child Health Care Hospital, Changzhou, China

**Keywords:** newborn screening, questionnaire, healthcare professionals, knowledge, attitude, newborn genetic screening

## Abstract

**Objectives:**

To understand the knowledge, attitude, willingness, and ability of healthcare professionals working in newborn screening (NBS) centers regarding newborn genetic screening (nGS).

**Methods:**

The questionnaire consisted of four sections with 27 questions and the data were collected by the WJX platform. All participants accessed the questionnaire by scanning a specific QR code with their mobile phones. Two researchers independently completed the summary and analysis.

**Results:**

A total of 258 valid questionnaires were collected from 43 NBS centers in six provinces of southeast China. In total, 209 (81.01%) participants were interested in nGS, and almost all participants (97.67%) thought that nGS was necessary in China. About 89.53% of participants thought that it could be used to effectively expand the diseases that could be screened, but 72.87% also worried about the inability to provide genetic counseling. About 55.34% suggested that nGS and tandem mass spectrometry (TMS) screening could be applied in a unite screening mode. The higher the institution and personal education levels, the higher the interest healthcare professionals displayed toward nGS. However, they also showed greater concern about the inability to provide genetic counseling and ethical issues. If a center had engaged in TMS screening, its staff would have been more likely to believe that nGS had great advantages. In addition, most participants had ethical concerns, such as “the psychological burden caused by carrying information regarding adult morbidity risk.”

**Conclusion:**

Most participants were interested and considered nGS necessary. The inability to provide genetic counseling may be the primary impediment to clinical practice. Three important influencing factors were level of education, institution level, and engagement in TMS screening.

## Introduction

Newborn screening (NBS) has been officially applied in clinical practice as an important public health project and has experienced continuous technological innovation and development (1). With the rapid advancement of DNA sequencing, newborn genetic screening (nGS) brings new opportunities to further expand neonatal genetic disease screening. In 2013, BabySeq (2), NBSeq (3), NC NEXUS (4), and STATseq (5) were launched successively to explore the application of genome sequencing in NBS. In China, nGS is rapidly gaining traction. Beijing (6), Shanghai (7), and Jiangsu (8) have successively conducted relevant studies, such as NESTS and NeoSeq. These studies have confirmed that nGS could further expand the genetic diseases that could not be found by traditional screening methods, provided more genetic information, and showed great technical advantages and broad prospects for application. It is considered as a great innovation in the field of NBS.

However, most scholars believe that many factors need full consideration before the clinical application becomes official (9, 10), such as technology, medical treatment, law, economy, ethics, psychology, and sociology. Currently, the issues needing attention are as follows: (1) which suitable technology should be selected for screening? There is no doubt that next-generation sequencing (NGS) is the appropriate technology platform. BabySeq (2), NBSeq (3), and NC NEXUS (4) selected whole-exome sequencing (WES) or exome sequencing (ES). They reported that the sensitivity and specificity were 60.0–88.6% and 93.7–100%, respectively. However, the high cost and technical difficulty of WES cannot be ignored. The study of NESTS (6) in China screened 596 types of serious genetic diseases through targeted sequencing, while NeoSeq used multiplex PCR amplicon sequencing (MTA-Seq) technology to screen 75 diseases (8). These technologies have certain advantages, and also have technical limitations. In short, the appropriate technologies for nGS are not unified. (2) How many diseases and pathogenic genes should be included in the scope of nGS? Although most scholars believed that screening diseases and pathogenic genes should follow certain principles (11), such as high incidence rate and the possibility for intervention or treatment. However, there is still no unified standard. (3) Can the ability to provide genetic counseling meet clinical requirements? It is well known that NGS technology can provide extensive genetic information, which poses great challenges to a clinical consultation. The relationship between this genetic information and future childhood health needs long-term observation. (4) What is the relationship between nGS and traditional NBS? How can both be integrated to achieve the best effect? Most scholars believe that nGS cannot replace traditional biochemical screening methods, but serves as an important supplement to them (12). However, there is still a lack of studies about the relationship between the two screening methods. (5) What is the reasonable population for nGS? Is it high-risk populations [such as NICU (13)] or the general newborn population (2)? Demonstrating this requires further study. (6) Other issues include ethical concerns, economic evaluation, recognition and acceptance of subjects, knowledge of medical staff, and psychosocial effect (14–17).

Understanding the knowledge, attitude, willingness, and ability of healthcare professionals working in NBS centers is of the utmost necessity before nGS officially enters clinical practice. Therefore, we designed a questionnaire and carried out a cross-sectional survey to evaluate and analyze relevant influencing factors.

## Materials and Methods

### Study Design

A cross-sectional survey of healthcare professionals working in NBS centers in southeast China was conducted online from 17 to 23 January 2022. In this study, a total of 258 valid questionnaires were collected from 43 NBS centers in six provinces of Southeast China (Supplementary Figure 1). Data were collected by the WJX platform (https://www.wjx.cn/). After the questionnaire was designed, a QR code was generated and promoted through WeChat (Tencent). All participants completed the questionnaires by scanning the specific QR code with their mobile phones. All participants were informed of the aim of the study before answering the questionnaire, voluntarily accepted the questionnaire, and completed it anonymously.

The study design and protocol were reviewed and approved by the ethics committee of Changzhou Maternal and Child Health Care Hospital [No. 2020 [70]].

### Questionnaires

The questionnaire was designed by the team at the Newborn Screening Center of Changzhou Maternal and Child Health Care Hospital, based on literature and previous clinical practice (8). The questionnaire consisted of four sections with 27 questions (Supplementary File 1). The Q1 section included demographic characteristics of participants (age, gender, education level, professional title, working years, professional field, and institution characteristics). The Q2 section contained questions about attitudes toward nGS, such as understanding, interest, and necessity. The Q3 section focused on the participants' knowledge of nGS technologies and requested their opinion on the principles for screening diseases, suitable technology for screening, reasonable modes of applying nGS and TMS screening, suitable populations for nGS, etc. The Q4 section included ethical concerns, cost, and promotion intention.

### Data Analysis

Data were derived from the WJX platform and collected using Excel 2010. Two researchers independently completed the summary and analysis. Data were analyzed using EmpowerStats (X&Y solutions, Inc.) and R (version 3.6.3) (18). Healthcare professionals were grouped according to their level of education, years worked, professional title, and professional field. Institutions were grouped according to level, properties, and size. The chi-square test was used to compare the differences between the groups. Odds ratio (OR) and 95% *CI*s were estimated by univariate regression analysis. Differences were considered statistically significant at a two-sided *p* of 0.05.

## Results

### Demographic Characteristics

The demographic characteristics of the participants are summarized in Table 1. First, 69.77% of institutions were maternal and child health hospitals and 95.74% were above-secondary hospitals. Additionally, 81.01% of NBS centers have engaged in TMS screening and the annual number of 63.95% of centers exceed 20,000. Second, about 83.33% of participants were women and 75.58% were over 30 years old. About 17.83% were clinicians in the NBS center, while 39.15% were laboratory technicians. Furthermore, 19.38 and 16.28% of participants engaged in dried blood spots (DBSs) collection and management, respectively.

**Table 1:** Demographic characteristics.

**Question**	***N*(%)**
**Question about institutions**	
**Q1-7: What is the level of your institution?**	
Tertiary general hospitals	52 (20.16)
Tertiary maternal and child health hospitals	127 (49.22)
Secondary general hospitals	15 (5.81)
Secondary maternal and child health care hospitals	53 (20.54)
Primary hospitals	5 (1.94)
Others	6 (2.33)
**Q1-8: Have you engaged in MS/MS screening program in your NBS center?**	
Yes	209 (81.01)
No	49 (18.99)
**Q1-9: How many newborns screen in your NBS center every year?**	
<20,000	93 (36.05)
20,000~50,000	113 (43.80)
50,000~100,000	50 (19.38)
>100,000	2 (0.78)
**Question about participants**	
**Q1-1: Your age?**	
<18	0 (0)
18~30	63 (24.42)
31~40	114 (44.19)
41~50	56 (21.71)
>50	25 (9.69)
**Q1-2: Your gender?**	
Male	43 (16.67)
Female	215 (83.33)
**Q1-3: Your level of education?**	
Junior middle school	0 (0)
Junior high school	8 (3.10)
Senior college	26 (10.08)
Undergraduate college	187 (72.48)
Masters	31 (12.02)
Doctorate	6 (2.33)
**Q1-4: Your professional title?**	
Primary title	92 (35.66)
Middle title	106 (41.09)
High title	57 (22.09)
Others	3 (1.16)
**Q1-5: How long have you worked in the newborn screening center?**	
<5	49 (18.99)
6~10	68 (26.36)
11~20	74 (28.68)
>20	67 (25.97)
**Q1-6: What is your professional field?**	
Clinician	46 (17.83)
Laboratory technician	101 (39.15)
Blood collection personnel	50 (19.38)
Management personnel	42 (16.28)
Others	19 (7.36)

### Attitudes Toward nGS

**Figure 1:**
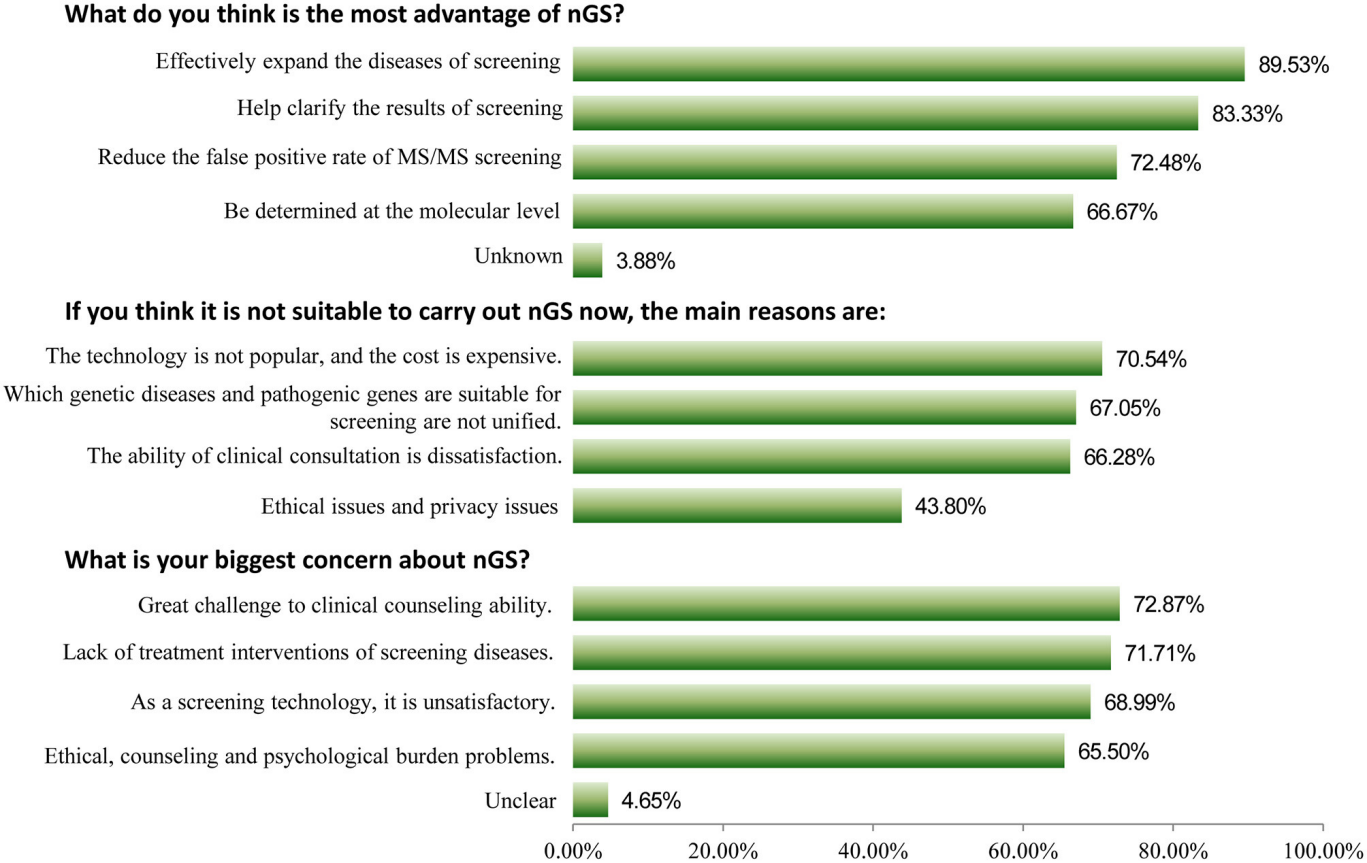
Three key issues about attitudes toward newborn genetic screening (nGS).

Among 258 participants, 209 (81.01%) indicated that they were interested in nGS, and 178 (69.00%) had a good awareness of it. Furthermore, 252 (97.67%) healthcare professionals considered nGS to be necessary for China, with only 5 participants considering it unnecessary. Three key issues were deeply analyzed, as shown in Figure 1. For the question “what do you think is the most advantage of nGS?,” 89.53% of participants thought that it could be used to effectively expand the diseases that could be screened, which were otherwise unsuitable for biochemical analysis or lacked reliable biomarkers. About 83.33% thought it could help clarify ambiguous or critical biochemical screening results, clarify the diagnosis, and guide accurate medication. About 72.48% thought that it could reduce the false positive rate of TMS screening. For the question “what is your biggest concern about nGS?,” the top three responses were “great challenge to clinical counseling ability because of too much genetic information (72.87%),” “lack of treatment interventions of screening diseases (71.71%),” and “as a screening technology, it is unsatisfactory, such as popularity, reporting time, and high cost (68.99%),” respectively. The primary reasons for it being unsuitable for screening were that “the technology is not popular, and the cost is expensive (70.54%),” “which genetic diseases and pathogenic genes are suitable for screening are not unified (67.05%),” and “the ability of clinical consultation is dissatisfaction (66.28%).”

### Knowledge of nGS

**Figure 2:**
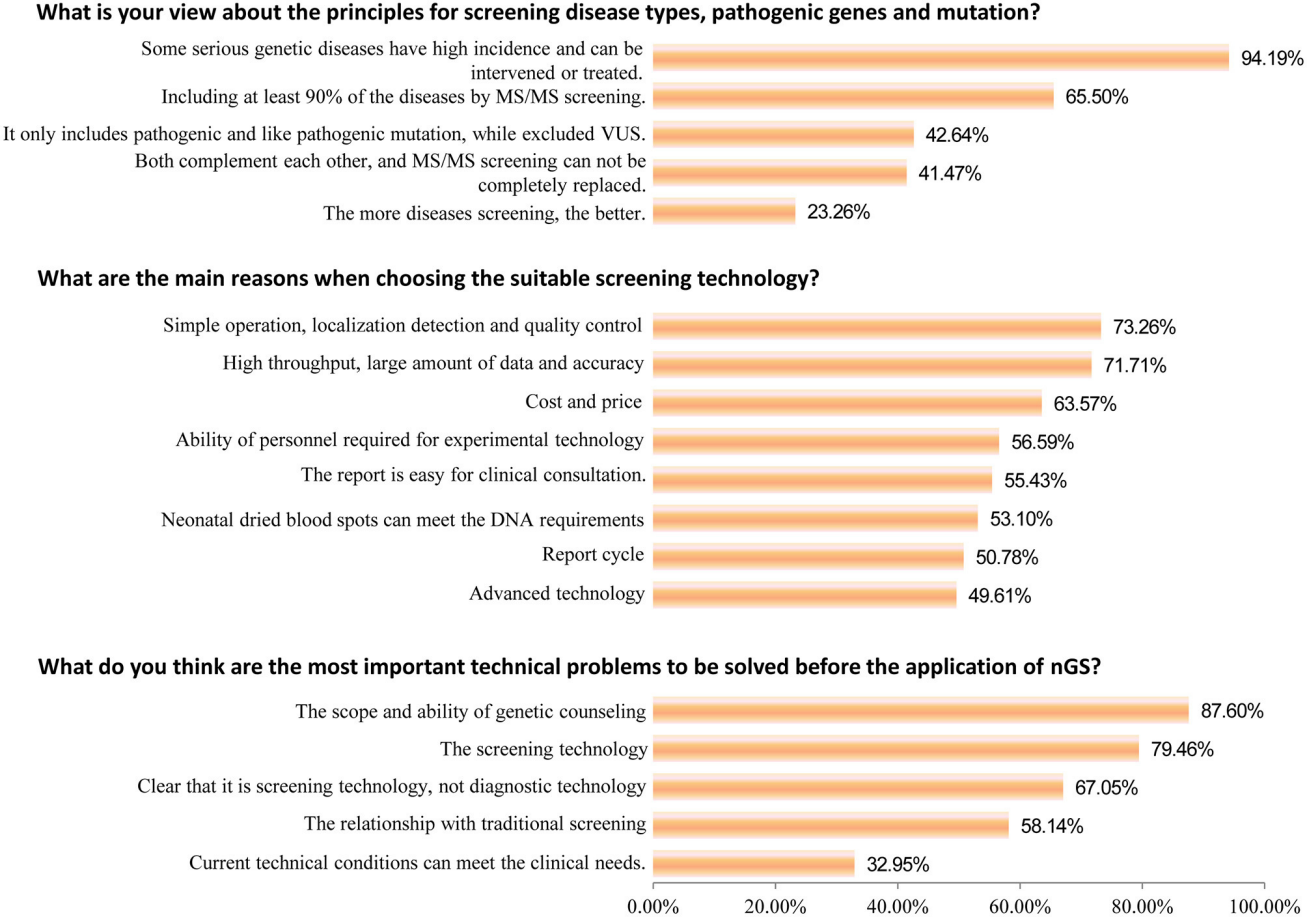
Knowledge of nGS (multiple options).

**Figure 3:**
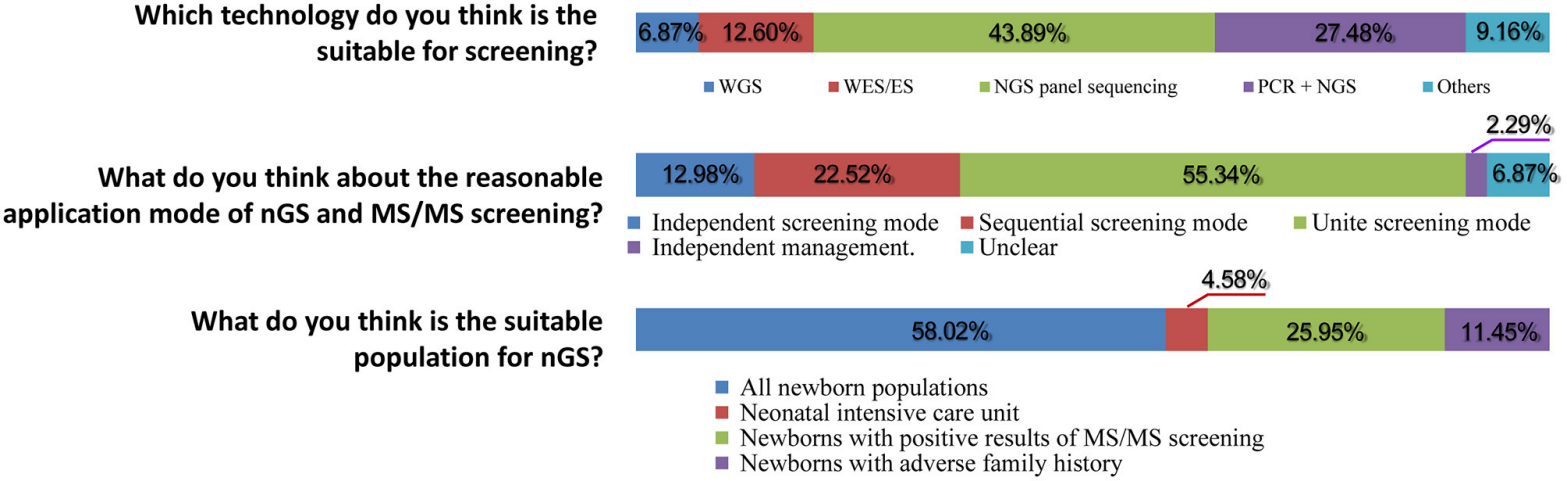
Knowledge of nGS (single choice).

Subsequently, important technical questions regarding nGS were investigated, such as screening principles, technology selection, and suitable population. First, the question of “the principles for screening disease types, pathogenic genes, and mutation” was discussed. As shown in Figures 2, 94.19% of participants suggested that certain serious genetic diseases with high incidence and the possibility of intervention or treatment could be included in nGS. More than half (65.50%) of healthcare professionals thought that nGS should include at least 90% of the diseases TMS screening currently covers. Only 23.26% of participants felt that the more diseases screened, the better the effect. Second, nGS panel sequencing was considered the most suitable technology, accounting for ~43.89% of participants, followed by PCR + NGS (27.48%). Surprisingly, only 6.87 and 12.60% of participants supported WGS and WES/ES as a suitable technology for nGS (Figure 3). The primary reasons for the selection of the above technology as suitable for screening are displayed in Figure 2. The top three reasons were “simple operation, localization detection, and quality control (73.26%),” “high throughput, a large amount of data and accuracy (71.71%),” and “cost and price (63.57%).” Third, most participants thought that there was a relationship between nGS and traditional TMS screening, while 2.29% thought that they were two independent methods (Figure 3). About 55.34% of healthcare professionals suggested that both methods could be applied in unite screening mode. In other words, both methods are conducted simultaneously, summarized, and analyzed together, and then judged and calculated. Additionally, the support rates of independent mode and sequential mode were 12.98% and 22.52%, respectively. Fourth, when asked about the suitable population for nGS, 58.02% of the subjects believed that all newborns were suitable for nGS. In contrast, only 4.58% believed that infants in the neonatal intensive care unit (NICU) were suitable for nGS (Figure 3). Fifth, most healthcare professionals considered many technical problems needing solutions before nGS could officially be put into clinical practice, especially “the scope and ability of genetic counseling (87.60%),” “the screening technology (79.46%),” and “clear that it is screening technology, not diagnostic technology (67.05%)” (Figure 2). Meanwhile, most healthcare professionals want to improve their ability in interpreting results and providing genetic counseling (83.97%) through symposiums, academic salons (90.08%), etc.

### Participant Characteristics Associated With Attitudes and Knowledge

**Figure 4:**
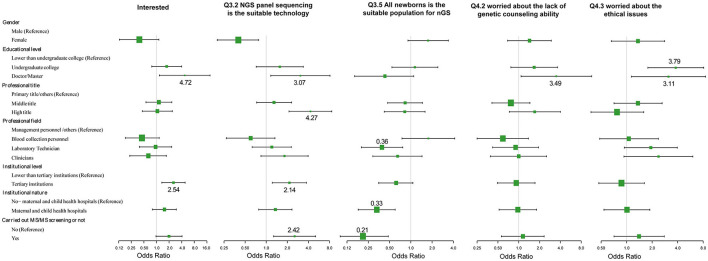
Association between demographic characteristics and attitudes and knowledge toward nGS.

The effects of demographic characteristics on the key issues of attitudes and knowledge toward nGS were analyzed (Figure 4; Supplementary Table 1). First, the level of education is a crucial factor. Healthcare professionals with higher education levels (doctorates and/or Masters) displayed greater interest in nGS (*OR* = 4.72, 95% *CI* 1.17~19.00, *p* = 0.0288), and considered the nGS panel as the suitable technical method currently (*OR* = 3.07, 95% *CI* 1.16~8.12, *p* = 0.0240). Additionally, they displayed greater concerns about the inability to provide genetic counseling (*OR* = 3.49, 95% *CI* 1.08~11.32, *p* = 0.0372) and ethical issues (*OR* = 3.11, 95% *CI* 1.14~8.52, *p* = 0.0273). In other words, they had greater risk awareness. Second, there were no significant differences in attitudes and knowledge between different professional fields in NBS centers. Surprisingly, the laboratory technicians were more cautious about the population nGS was being conducted on. They seemed to disapprove of the direct use of nGS in all neonatal populations (*OR* = 0.39, 95% *CI* 0.20~0.77, *p* = 0.0061). Third, the institution level also affected the attitude and knowledge of participants. The higher the institution level, the more interested the personnel were in nGS (*OR* = 2.54, 95% *CI* 1.34~4.82, *p* = 0.0044), and the clearer the choice of appropriate technology (*OR* = 2.14, 95% *CI* 1.22~3.75, *p* = 0.0083). However, there was little association with the nature of the institution, i.e., whether it was a maternal and child health institution or not. Fourth, the attitudes and knowledge of healthcare professionals were related to the center engaging in TMS screening. If the center had engaged in TMS screening, the staff would be more likely to believe that nGS had great advantages (*OR* = 2.42, 95% *CI* 1.05~5.58, *p* = 0.0375). nGS can screen diseases that are not suitable for biochemical analysis or do not have reliable biomarkers, and can thus be used to effectively expand the scope of screening. However, these staff also displayed greater caution about the population being screened (*OR* = 0.21, 95% *CI* 0.10~0.48, *p* = 0.0002).

### Other Findings

Almost all healthcare professionals (96.56%) were willing to promote nGS. However, they believed that their lack of ability, specifically in genetic counseling, would restrict the effect of their promotion produced. Most participants also had ethical concerns. The top three ethical concerns were “psychological burden caused by the carrying information of adult morbidity risk (74.81%),” “privacy of gene information (73.28%),” and “there may be misunderstanding and interpretation of genetic information and cause harm (67.56%).” Finally, 47.33% of participants suggested that the price of nGS be <500 Yuan, and 40.46% suggested that it be between 500 and 1,000 Yuan.

## Discussion

Newborn genetic screening as a research field is in its nascency, encompassing elements, such as technology, strategy, population, safety, and ethics. Therefore, the necessity of fully understanding the knowledge, attitude, willingness, and abilities of service objects (parents) and healthcare professionals before an application is paramount. Relevant literature (Banyseq, etc.) indicates that some questionnaires have been administered abroad. However, there have been no such studies in China. Concurrently, recent reports have mainly been aimed at the parents of newborns, and few focused on healthcare professionals. For example, Waisbren's group (19) surveyed 514 parents to determine interest in newborn genomic testing. The different degrees of interest in nGS were extremely (18.1%), very (28.0%), somewhat (36.6%), a little (10.9%), and not at all (6.4%). The results showed that the parents of healthy newborns were highly interested in nGS. Similarly, Goldenberg's group (20) reported that 74% of parents were somewhat interested in utilizing nGS, and mainly focused on the test accuracy and its ability to prevent diseases. Furthermore, Genetti et al. (21) postulated that the main reasons for the decline of interest from parents were privacy/insurability (41%) and uncertain/unfavorable results (23%). A retrospective survey on parents of infants in the NICU showed that most subjects had positive feelings about nGS and 100% felt that it was generally beneficial. In addition, they showed an understanding of the psychological risks involved (22). These studies showed that most parents had a positive attitude toward nGS and thought it would contribute to the health of newborns. However, their concerns and psychological stress cannot be ignored.

Newborns and their parents are the beneficiaries of nGS services, and they are greatly influenced by clinicians. Meanwhile, healthcare professionals are the primary executors of nGS. Their knowledge, attitude, and willingness directly affect the implementation and promotion of the procedure. However, few studies exist regarding healthcare professionals. Pereira et al. (23) explored parents' and clinicians' attitudes, respectively, and found that 71% of parents and 51% of clinicians believed nGS would be beneficial to health. However, compared with parents, clinicians paid more attention to the risks of nGS and endorsed concerns about privacy and discrimination related to genetic information. In this study, we conducted a cross-sectional survey to understand the knowledge, attitude, willingness, and ability of healthcare professionals working in NBS centers. We found that 81.01% of participants showed interest in nGS and almost everyone considered nGS to be necessary for China. Our result is significantly higher than the study of Pereira et al. (23), which may be because this study was directed at the staff in NBS centers, who should have a deeper understanding of the significance of neonatal disease screening. They believed that the greatest advantage of nGS was that it could be used to effectively expand the diseases being screened, which were otherwise unsuitable for biochemical analysis or lacked reliable biomarkers. Meanwhile, 72.87% of participants also worried about the inability to provide genetic counseling and believed it would be the main impediment to clinical practice. Additionally, 94.19% of healthcare professionals thought that nGS should follow strict screening principles for diseases and pathogenic genes, and suggested certain serious genetic diseases with high incidence and a possibility for intervention or treatment which could be screened. This is consistent with Bayseq (11). Chinese healthcare professionals prefer the NGS panel over WES as a suitable screening technology. The main reasons for this were the technical difficulty and price. Integrating nGS with the traditional screening makes for an interesting discussion. Approximately 55.34% of participants suggested that both methods could be applied in unite screening mode, summarized and analyzed together, and judged, and the results could then be calculated. This provides some direction for the clinical practice of nGS and TMS screening in the future. Only 4.58% participants believed that the infants in NICU were suitable for nGS, while most people thought it could be directly used for all newborn populations, which countered our expectations. Ceyhan-Birsoy's group (2) presented 159 newborns (32 from NICU and 127 healthy newborns) in the BabySeq Project. A total of 15 newborns were identified with genetic variations that conferred disease risk, including 10 (7.87%) healthy newborns and 5 cases (14.29%) from the NICU. Newborns from the NICU were reconfirmed as population at high risk for genetic diseases, and it seems that they are in greater need of nGS. However, we should remind ourselves that the genetic diseases NICU newborns deal with have increased complexity. Is the technology based on NGS panel enough, or is the effect of WES technology better? This suggests that different screening techniques might be selected for nGS in different populations. However, the current scope of clinical practice is still very limited, and requires deeper study.

Next, we discuss the relationship between the demographic characteristics of healthcare professionals and their attitude toward and knowledge of nGS. Three important influencing factors were found: level of education, institution level, and engagement in TMS screening. Overall, the higher the institutional level and personal education level, the more interested the healthcare professionals will be toward nGS, and the clearer the selection of technology. However, their risk awareness was also higher. They frequently displayed increased concern about the risk caused by their own inability and ethical concerns. Concurrently, when the NBS center had engaged in TMS screening, its staff displayed more experience and were clearer about the advantages and objectives of nGS. They were also more cautious about the screening population. This study, focusing on influencing factors, is obviously insufficient prior to large-scale application of nGS. Future studies can help us adjust in time and prepare for the implementation of the procedure.

This study is, to our knowledge, the first cross-sectional survey on the knowledge, attitude, willingness, and ability of healthcare professionals working in NBS centers regarding nGS. Nevertheless, this study has some limitations. It is uncertain how representative our sample is of NBS centers in China. The study included 43 centers in six provinces. However, the number of valid questionnaires collected in some provinces were insufficient (<10), and we did not include them in data analysis. The content of the questionnaire was based on relevant literature and our own inferences, and although its validity has been demonstrated to some extent, it may require further optimization.

In conclusion, we carried out a cross-sectional survey to understand the knowledge, attitude, willingness, and ability of healthcare professionals working in NBS centers regarding nGS. Most participants were interested and considered nGS necessary. They already had knowledge and risk awareness. The inability to provide genetic counseling may be the primaryimpediment to clinical practice. Three important influencing factors were level of education, institution level, and engagement in TMS screening.

## Data Availability Statement

The original contributions presented in the study are included in the article/Supplementary Material, further inquiries can be directed to the corresponding author/s.

## Ethics Statement

The study design and protocol were reviewed and approved by the Ethics Committee of Changzhou Maternal and Child Health Care Hospital [NO.2020[70]]. Written informed consent for participation was not required for this study in accordance with the national legislation and the institutional requirements.

## Author Contributions

BY and LZ conducted the questionnaire and participated in the study design. XW, YY, LZ, and BY conducted clinical consultations and laboratory tests. WL and BY performed the statistical analysis. BY conceived the study, participated in its design and coordination, and helped draft the manuscript. All authors contributed to the article and approved the submitted version.

## Funding

This study was funded by the Jiangsu Maternal and Children Health Care Key Discipline (2021).

## Conflict of Interest

The authors declare that the research was conducted in the absence of any commercial or financial relationships that could be construed as a potential conflict of interest.

## Publisher's Note

All claims expressed in this article are solely those of the authors and do not necessarily represent those of their affiliated organizations, or those of the publisher, the editors and the reviewers. Any product that may be evaluated in this article, or claim that may be made by its manufacturer, is not guaranteed or endorsed by the publisher.
