# Supplementary material for: Are We Ready for Newborn Genetic Screening? A Cross-Sectional Survey of Healthcare Professionals in Southeast China
Source: Front Pediatr. 2022 May 6;10:875229. doi: 10.3389/fped.2022.875229 (PMC9120836; doi:10.3389/fped.2022.875229)
Supplement: Supplementary file 1 [file Data_Sheet_1.docx]

**Supplementary file 1**

**Questionnaire survey for newborn genomic sequencing**

**(Newborn Screening Center version)**

Newborn genomic screening (nGS) is a new research area, and shows the prospect of broad applications. Many scholars believe that many factors should be fully considered before it enters the clinic, including technology, medical treatment, law, economy, ethics, psychology and sociology.

You have been engaged in newborn screening for a long time. In order to actively prepare for the clinical promotion project, could you take a few minutes to carefully complete the following questionnaire to guide our future work direction？ Thank you!

The questionnaire was completed anonymously, following the principles of voluntariness and confidentiality.

**Q1：Demographics**

**Q1-1: You age?**

1. <18
2. 18~25
3. 26~30
4. 31~35
5. 36~40
6. 41~45
7. 46~50
8. >50

**Q1-2: Your gender?**

1. Male
2. Femal

**Q1-3: Your educational level?**

1. Junior middle school
2. Junior high school
3. Senior College
4. Undergraduate college
5. Master
6. Doctor

**Q1-4: Your professional title?**

1. Primary title
2. Middle title
3. High title
4. Others

**Q1-5: How long have you worked in newborn screening center?**

1. <5
2. 6~10
3. 11~20
4. >20

**Q1-6: What is your professional field?**

A. Clinicians

B. Laboratory Technician

C. Blood collection personnel

D. Management personnel

**Q1-7: What is the level of your institution?**

1. Tertiary general hospitals
2. Tertiary maternal and child health hospitals
3. Secondary general hospitals
4. Secondary maternal and child health care hospitals
5. Primary hospitals
6. Others

**Q1-8: Have you carried out MS / MS screening program in your NBS center?**

1. Yes
2. No

**Q1-9: How many newborn screening in your NBS center every year?**

1. <20000
2. 20000~50000
3. 50000~100000
4. >100000

**Q2：Attitudes**

**Q2-1: Do you know about Newborn genomic screening?**

1. Unknown
2. Know a little
3. Know some
4. Know many

**Q2-2: Do you interested in Newborn genomic screening?**

1. Uninterested
2. A little
3. Some
4. Very much

**Q2-3: Do you think it is necessary to carry out Newborn genomic screening in China?**

1. Very much
2. More
3. Unnecessary
4. Unclear

**Q2-4: If you think it is not suitable to carry out nGS now, the main reasons are:**

1. Which genetic diseases and pathogenic genes are suitable for screening are not unified.
2. The technology is not popular, and the cost is expensive.
3. The ability of clinical consultation is dissatisfaction.
4. Ethical issues and privacy issues.

**Q2-5: What do you think is the most advantage of nGS?**

1. The source of mutation can be determined at the molecular level.
2. NBS can be extended to those diseases that are not suitable for biochemical analysis or do not have reliable biomarkers, so as to further effectively expand the scope of screening.
3. It can help clarify ambiguous or critical biochemical screening results, clarify diagnosis, and guide accurate medication.
4. It can help reduce the false positive rate of MS / MS screening.
5. Unknown.

**Q2-6: What is your biggest concern about nGS?**

1. Lack of treatment interventions of screening diseases.
2. Great challenge to clinical counseling ability because of too much genetic information.
3. Too much genetic information may cause ethical, counseling and psychological burden problems.
4. As a screening technology, it is unsatisfactory, such as popularity, reporting time, high cost.
5. Unclear

**Q3：Knowledge**

**Q3-1: What is your view about the principles for screening disease types, pathogenic genes and mutation?**

1. The more diseases screening, the better.
2. Some serious genetic diseases have high incidence and can be intervened or treated.
3. It only includes pathogenic and like pathogenic mutation, while excluded VUS.
4. Including at least 90% of the diseases by MS/MS screening.
5. Both complement each other, and MS/MS screening can not be completely replaced.

**Q3-2: Which technology do you think is the suitable for screening?**

1. WGS
2. WES/ES
3. NGS panel sequencing
4. PCR + NGS
5. Others

**Q3-3: What are the main reasons when choosing the suitable screening technology?**

1. High throughput, large amount of data and accuracy
2. Simple operation, localization detection and quality control
3. Ability of personnel required for experimental technology
4. Cost and price
5. Report cycle
6. Advanced technology
7. Neonatal dried blood spots can meet the DNA requirements
8. The report is easy for clinical consultation.

**Q3-4: What do you think about the reasonable application mode of nGS and MS/MS screening?**

1. Independent screening mode: both completely independent, the results are compared and summarized.
2. Sequential screening mode: traditional screening is carried out firstly, and nGS is carried out for suspected positive.
3. Unite screening mode: Both test at the same time, summarize the results and recall for diagnosis.
4. It doesn't matter. As two independent methods, they are independent management.
5. Unclear

**Q3-5: At present, what do you think is the suitable population for nGS?**

1. All newborn populations
2. Neonatal intensive care unit
3. Newborns with positive results of MS/MS screening
4. Newborns with adverse family history

**Q3-6: What capabilities do you think need to be supplemented most in order to clinical application?**

1. Knowledge of neonatal genetic diseases, such as diagnosis, treatment and intervention
2. Abilities of report interpretation and genetic counseling
3. Abilities of Experimental operation and data analysis
4. Study progress
5. Policies and regulations, technical specifications, expert consensus, etc
6. No, current capacity has met the needs

**Q3-7: How do you want to improve the above capabilities?**

1. Academic conferences and education projects
2. Symposium, academic Salon, case discussion, multi center exchange
3. Network platform
4. Literature

**Q3-8: What do you think are the most important technical problems to be solved before the application of nGS?**

1. The screening technology
2. The relationship with traditional screening
3. Clear that it is screening technology, not diagnostic technology
4. The scope and ability of genetic counseling
5. Current technical conditions can meet the clinical needs.

**Q4：Others**

**Q4-1: Are you willing to promote nGS?**

1. Yes
2. No

**Q4-2: What do you think is the main reason that hinders the promotion of nGS？**

1. The technology is not mature and cannot be used for large-scale screening.
2. Healthcare professionals are reluctant to promote because of lack of abilities.
3. The cost is too expensive.
4. The diseases which are screened can’t be effectively treated and intervented.
5. The genetic counseling ability and relevant supporting policies can not be met.
6. It doesn't have any clinical value.

**Q4-3: What do you think are the main ethical problems of nGS?**

1. Privacy of gene information
2. Children may be discriminated against because of gene information.
3. Psychological burden caused by the carrying information of adult morbidity risk
4. Genotype phenotype association, penetrance, etc
5. There may be misunderstanding and interpretation of genetic information and cause harm.
6. No problem

**Q4-4: What do you think is the reasonable price?**

1. < CNY 500
2. CNY 500~1000
3. CNY 1000~2000
4. > CNY 2000
