# Supplementary material for: Are We Ready for Newborn Genetic Screening? A Cross-Sectional Survey of Healthcare Professionals in Southeast China
Source: Front Pediatr. 2022 May 6;10:875229. doi: 10.3389/fped.2022.875229 (PMC9120836; doi:10.3389/fped.2022.875229)
Supplement: Supplementary file 2 [file Data_Sheet_2.docx]

**Supplementary figure 1. The distribution map of the cross-sectional survey**

**
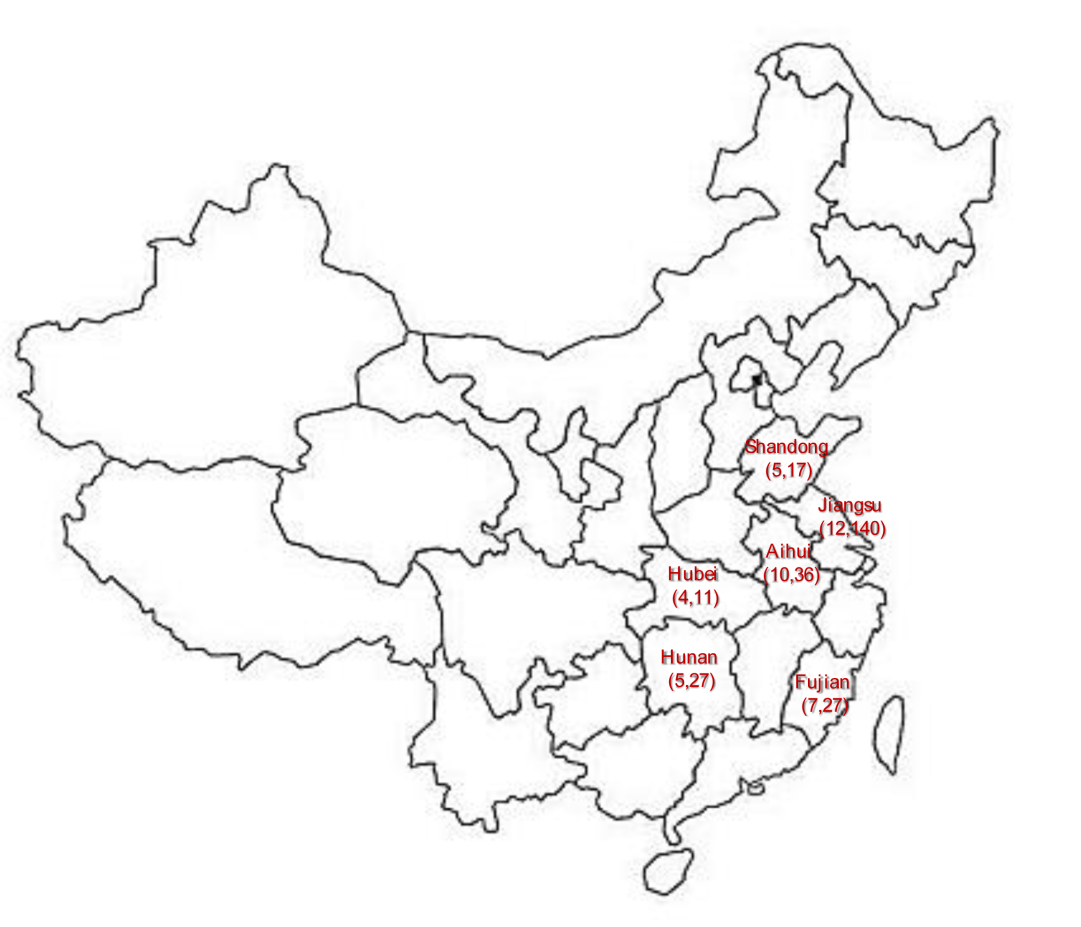
**

Note: Province (centers, participants)
