# Supplementary material for: Are We Ready for Newborn Genetic Screening? A Cross-Sectional Survey of Healthcare Professionals in Southeast China
Source: Front Pediatr. 2022 May 6;10:875229. doi: 10.3389/fped.2022.875229 (PMC9120836; doi:10.3389/fped.2022.875229)
Supplement: Supplementary file 3 [file Table_1.docx]

**Supplementary table 1. The effects of demographic characteristics on attitude and knowledge towards nGS** [OR (95%CI) Pvalue]

|  | **Statistics** | **Understand** | **Interested** | **Q2-4** | **Q2-5** | **Q2-6** | **Q3-1** | **Q3-2** | **Q3-4** | **Q3-5** | **Q4-2** | **Q4-3** |
| --- | --- | --- | --- | --- | --- | --- | --- | --- | --- | --- | --- | --- |
| **Gender** | | | | | | | | | | | | |
| Male | 43 (16.67%) | Reference | | | | | | | | | | |
| Female | 215 (83.33%) | 0.96 (0.47, 1.95) 0.9042 | 0.39 (0.13, 1.14) 0.0853 | 0.64 (0.30, 1.38) 0.2538 | 1.00 (0.36, 2.78) 1.0000 | 0.71 (0.33, 1.54) 0.3903 | 1.37 (0.43, 4.34) 0.5953 | 0.40 (0.20, 0.78) 0.0071 | 0.70 (0.33, 1.48) 0.3521 | 1.76 (0.91, 3.40) 0.0929 | 1.43 (0.69, 2.95) 0.3327 | 1.36 (0.66, 2.80) 0.4056 |
| **Educational level** | | | | | | | | | | | | |
| Lower than undergraduate college | 34 (13.18%) | Reference | | | | | | | | | | |
| Undergraduate college | 187 (72.48%) | 2.19 (1.04, 4.61) 0.0390 | 1.75 (0.77, 3.98) 0.1833 | 1.04 (0.47, 2.27) 0.9263 | 2.16 (0.84, 5.61) 0.1118 | 1.00 (0.45, 2.23) 1.0000 | 1.53 (0.47, 4.92) 0.4766 | 1.56 (0.72, 3.39) 0.2583 | 0.53 (0.24, 1.19) 0.1244 | 1.14 (0.54, 2.41) 0.7237 | 1.67 (0.77, 3.64) 0.1954 | 3.79 (1.78, 8.11) 0.0006 |
| Doctor/Master | 37 (14.34%) | 2.40 (0.89, 6.46) 0.0830 | 4.72 (1.17, 19.00) 0.0288 | 1.49 (0.53, 4.21) 0.4536 | 2.94 (0.69, 12.45) 0.1434 | 1.30 (0.45, 3.71) 0.6290 | inf. (0.00, Inf) 0.9877 | 3.07 (1.16, 8.12) 0.0240 | 0.67 (0.24, 1.90) 0.4536 | 0.43 (0.16, 1.10) 0.0793 | 3.49 (1.08, 11.32) 0.0372 | 3.11 (1.14, 8.52) 0.0273 |
| **Professional title** | | | | | | | | | | | | |
| Primary title/others | 95 (36.82%) | Reference | | | | | | | | | | |
| Middle title | 106 (41.09%) | 0.92 (0.51, 1.69) 0.7985 | 1.14 (0.56, 2.32) 0.7076 | 1.41 (0.79, 2.54) 0.2497 | 0.86 (0.37, 2.00) 0.7270 | 1.16 (0.63, 2.16) 0.6293 | 0.73 (0.25, 2.12) 0.5594 | 1.29 (0.73, 2.30) 0.3839 | 0.87 (0.45, 1.66) 0.6635 | 0.83 (0.47, 1.46) 0.5226 | 0.77 (0.41, 1.45) 0.4220 | 1.36 (0.71, 2.62) 0.3533 |
| High title | 57 (22.09%) | 0.84 (0.41, 1.69) 0.6183 | 1.05 (0.46, 2.39) 0.9162 | 2.55 (1.17, 5.55) 0.0182 | 1.36 (0.45, 4.14) 0.5862 | 0.91 (0.44, 1.84) 0.7845 | 0.89 (0.24, 3.31) 0.8659 | 4.27 (2.11, 8.61) <0.0001 | 0.63 (0.28, 1.44) 0.2712 | 0.82 (0.42, 1.59) 0.5510 | 1.70 (0.73, 4.00) 0.2207 | 0.77 (0.38, 1.59) 0.4860 |
| **Professional field** | | | | | | | | | | | | |
| Management personnel /others | 61 (23.64%) | Reference | | | | | | | | | | |
| Blood collection personnel | 50 (19.38%) | 1.29 (0.53, 3.11) 0.5717 | 0.45 (0.18, 1.16) 0.0994 | 0.63 (0.29, 1.36) 0.2421 | 0.60 (0.21, 1.75) 0.3514 | 0.55 (0.24, 1.22) 0.1398 | 1.52 (0.42, 5.52) 0.5255 | 0.60 (0.27, 1.33) 0.2069 | 1.11 (0.43, 2.89) 0.8248 | 1.77 (0.75, 4.18) 0.1908 | 0.59 (0.25, 1.38) 0.2229 | 1.06 (0.48, 2.37) 0.8820 |
| Laboratory Technician | 101 (39.15%) | 0.52 (0.26, 1.04) 0.0647 | 0.95 (0.39, 2.30) 0.9071 | 1.42 (0.72, 2.83) 0.3134 | 1.79 (0.60, 5.39) 0.2985 | 1.59 (0.74, 3.39) 0.2320 | 2.56 (0.78, 8.47) 0.1229 | 1.20 (0.63, 2.28) 0.5854 | 1.94 (0.89, 4.24) 0.0939 | 0.39 (0.20, 0.77) 0.0061 | 0.90 (0.42, 1.93) 0.7851 | 1.93 (0.94, 3.96) 0.0738 |
| Clinicians | 46 (17.83%) | 1.16 (0.48, 2.81) 0.7472 | 0.64 (0.23, 1.72) 0.3722 | 3.00 (1.14, 7.86) 0.0255 | 0.74 (0.24, 2.27) 0.5935 | 0.52 (0.23, 1.17) 0.1144 | 1.89 (0.46, 7.76) 0.3754 | 1.82 (0.84, 3.96) 0.1307 | 0.67 (0.23, 1.97) 0.4639 | 0.65 (0.29, 1.44) 0.2867 | 1.00 (0.39, 2.53) 0.9928 | 2.38 (0.93, 6.03) 0.0690 |
| **Institutional level** | | | | | | | | | | | | |
| Lower than tertiary institutions | 77 (29.84%) | Reference | | | | | | | | | | |
| Tertiary institutions | 181 (70.16%) | 1.42 (0.81, 2.49) 0.2261 | 2.54 (1.34, 4.82) 0.0044 | 1.87 (1.06, 3.28) 0.0295 | 1.67 (0.76, 3.67) 0.1994 | 0.99 (0.55, 1.79) 0.9795 | 2.26 (0.88, 5.81) 0.0897 | 2.14 (1.22, 3.75) 0.0083 | 1.45 (0.74, 2.83) 0.2823 | 0.62 (0.35, 1.07) 0.0869 | 0.92 (0.49, 1.72) 0.7994 | 0.87 (0.47, 1.62) 0.6611 |
| **Institutional nature** | | | | | | | | | | | | |
| No- maternal and child health hospitals | 75 (29.07%) | Reference | | | | | | | | | | |
| Maternal and child health hospitals | 183 (70.93%) | 0.68 (0.37, 1.24) 0.2086 | 1.55 (0.80, 2.98) 0.1913 | 1.30 (0.73, 2.31) 0.3673 | 1.25 (0.56, 2.82) 0.5849 | 0.72 (0.39, 1.33) 0.2883 | 1.87 (0.72, 4.84) 0.1993 | 1.35 (0.78, 2.33) 0.2882 | 0.99 (0.52, 1.87) 0.9634 | 0.33 (0.18, 0.60) 0.0003 | 0.97 (0.52, 1.81) 0.9202 | 1.01 (0.54, 1.88) 0.9736 |
| **Carried out MS/MS screening or not** | | | | | | | | | | | | |
| No | 49 (18.99%) | Reference | | | | | | | | | | |
| Yes | 209 (81.01%) | 1.38 (0.72, 2.64) 0.3367 | 1.99 (0.97, 4.08) 0.0607 | 1.75 (0.92, 3.34) 0.0877 | 2.42 (1.05, 5.58) 0.0375 | 1.77 (0.92, 3.40) 0.0850 | 1.58 (0.54, 4.62) 0.4013 | 2.54 (1.27, 5.06) 0.0081 | 1.36 (0.62, 3.00) 0.4448 | 0.21 (0.10, 0.48) 0.0002 | 1.15 (0.56, 2.33) 0.7023 | 1.40 (0.71, 2.78) 0.3331 |

Note:

**Q2-4: If you think it is not suitable to carry out nGS now, the main reason is “**The technology is not popular, and the cost is expensive.**”**

**Q2-5: What do you think is the most advantage of nGS?** NBS can be extended to those diseases that are not suitable for biochemical analysis or do not have reliable biomarkers, so as to further effectively expand the scope of screening.

**Q2-6: What is your biggest concern about nGS?** Great challenge to clinical counseling ability because of too much genetic information.

**Q3-1: What is your view about the principles for screening disease types, pathogenic genes and mutation?** Some serious genetic diseases have high incidence and can be intervened or treated.

**Q3-2: Which technology do you think is the suitable for screening?** NGS panel sequencing

**Q3-4: What do you think about the reasonable application mode of nGS and MS/MS screening?** Unite screening mode: Both test at the same time, summarize the results and recall for diagnosis.

**Q3-5: At present, what do you think is the suitable population for nGS?** All newborn populations.

**Q4-2: What do you think is the main reason that hinders the promotion of nGS？**The genetic counseling ability and relevant supporting policies can not be met.

**Q4-3: What do you think are the main ethical problems of nGS?** Psychological burden caused by the carrying information of adult morbidity risk.
